# Supplementary material for: The Effect of Psychological Distress on Measurement Invariance in Measures of Mental Wellbeing
Source: Int J Environ Res Public Health. 2022 Aug 15;19(16):10072. doi: 10.3390/ijerph191610072 (PMC9408727; doi:10.3390/ijerph191610072)
Supplement: Supplementary file 1 [file ijerph-19-10072-s001.zip › ijerph-1830175-supplementary.pdf]

Table S1. Results of measurement invariance testing in the original MHC-SF factor structure

Model	χ^2	RMSEA	CFI	TLI	$\Delta\chi^2$
Configural	4596.5 (148)	0.064	0.913	0.89	43.66
Metric	4640.2 (159)	0.062	0.913	0.90	$p < 0.0001$

Table S2. Identification of the source of metric invariance in the original MHC-SF factor structure.

Relaxed item:	χ^2	df	$\Delta\chi^2$	Δdf	P
1	4638.7	158	1.5	1	0.221
2	4622.2	158	18	1	< .001*
3	4631.9	158	8.3	1	0.004*
4	4636.1	158	4.1	1	0.043*
5	4640	158	0.2	1	0.655
6	4635.9	158	4.3	1	0.038*
7	4639.7	158	0.5	1	0.480
8	4640.2	158	0	1	1.00
9	4640.2	158	0	1	1.000
10	4639.7	158	0.5	1	0.480
11	4639.1	158	1.1	1	0.294
12	4627.1	158	13.1	1	< .001*
13	4640.2	158	0	1	1.00
14	4634.3	158	5.9	1	0.015*
